# Supplementary material for: Changes in Eating Behaviors and Their Associations with Weight Loss in Japanese Patients Who Underwent Laparoscopic Sleeve Gastrectomy
Source: Nutrients. 2023 Jan 10;15(2):353. doi: 10.3390/nu15020353 (PMC9866351; doi:10.3390/nu15020353)
Supplement: Supplementary file 1 [file nutrients-15-00353-s001.zip › nutrients-2140223-supplementary.pdf]

Table S1. Correlations between changes in eating behaviors from before to 12 months after surgery and %TWL at 12 months after surgery.

Changes in eating behaviors (From before to 12 months after surgery)	Unadjusted		Sex, age, and pre-operative BMI -adjusted	
	R	*P* value	Std β	*P* value
Δ Recognition for weight and constitution	-0.10	0.508	-0.07	0.654
Δ External eating behavior	-0.03	0.855	-0.03	0.865
Δ Emotional eating behavior	-0.19	0.200	-0.18	0.248
ΔSense of hunger	-0.14	0.340	-0.12	0.412
Δ Eating style	-0.19	0.200	-0.20	0.173
Δ Food preference	-0.06	0.689	0.05	0.766
Δ Regularity of eating habits	-0.05	0.748	-0.08	0.606
Δ Total score	-0.11	0.445	-0.12	0.462

Abbreviations: %TWL, the percent total weight loss; BMI, body mass index.

Table S2. Correlations between changes in eating behaviors from before to 12 months after surgery and %EWL at 12 months after surgery.

**Changes in eating behaviors**	**Unadjusted**		**Sex, age, and pre-operative BMI -adjusted**	
**(From before to 12 months after surgery)**	**R**	***P* value**	**Std β**	***P* value**
**Δ Recognition for weight and constitution**	**0.04**	**0.770**	**0.05**	**0.722**
**Δ External eating behavior**	**0.09**	**0.530**	**0.02**	**0.873**
**Δ Emotional eating behavior**	**-0.03**	**0.845**	**-0.15**	**0.329**
**ΔSense of hunger**	**-0.05**	**0.729**	**-0.12**	**0.421**
**Δ Eating style**	**-0.18**	**0.223**	**-0.23**	**0.113**
**Δ Food preference**	**0.08**	**0.606**	**0.002**	**0.990**
**Δ Regularity of eating habits**	**-0.08**	**0.599**	**-0.10**	**0.483**
**Δ Total score**	**0.02**	**0.898**	**-0.08**	**0.604**

Abbreviations: %EWL, the percent excess weight loss; BMI, body mass index.

Table S3. Differences in clinical parameters at the first visit between patients who underwent metabolic and bariatric surgery and those who did not.

Clinical parameters	Operative	Non-operative	*p* value
**Sex (male/female)**	**30/27**	**13/18**	**0.378**
**Age (years)**	**44.4 ± 8.6**	**43.2 ± 10.9**	**0.575**
**BW (kg)**	**117.7 ± 25.1**	**120.4 ± 21.1**	**0.616**
**BMI (kg/m^2)**	**41.7 ± 7.9**	**45.2 ± 7.1**	**0.042**
**Eating behaviors at the first visit**			
**Recognition for weight and constitution (%)**	**66.4 ± 13.4**	**73.0 ± 12.4**	**0.026**
**External eating behavior (%)**	**58.7 ± 16.0**	**58.5 ± 20.8**	**0.959**
**Emotional eating behavior (%)**	**44.2 ± 16.9**	**53.7 ± 21.0**	**0.026**
**Sense of hunger (%)**	**59.1 ± 14.5**	**60.8 ± 17.8**	**0.651**
**Eating style (%)**	**64.8 ± 20.4**	**64.8 ± 24.0**	**0.996**
**Food preference (%)**	**60.4 ± 18.0**	**57.5 ± 17.6**	**0.486**
**Regularity of eating habit (%)**	**61.0 ± 15.5**	**60.6 ± 18.8**	**0.915**
**Total score (%)**	**60.0 ± 12.0**	**61.2 ± 15.1**	**0.698**

Data are presented as means (± SD). BW, body weight; BMI, body mass index.

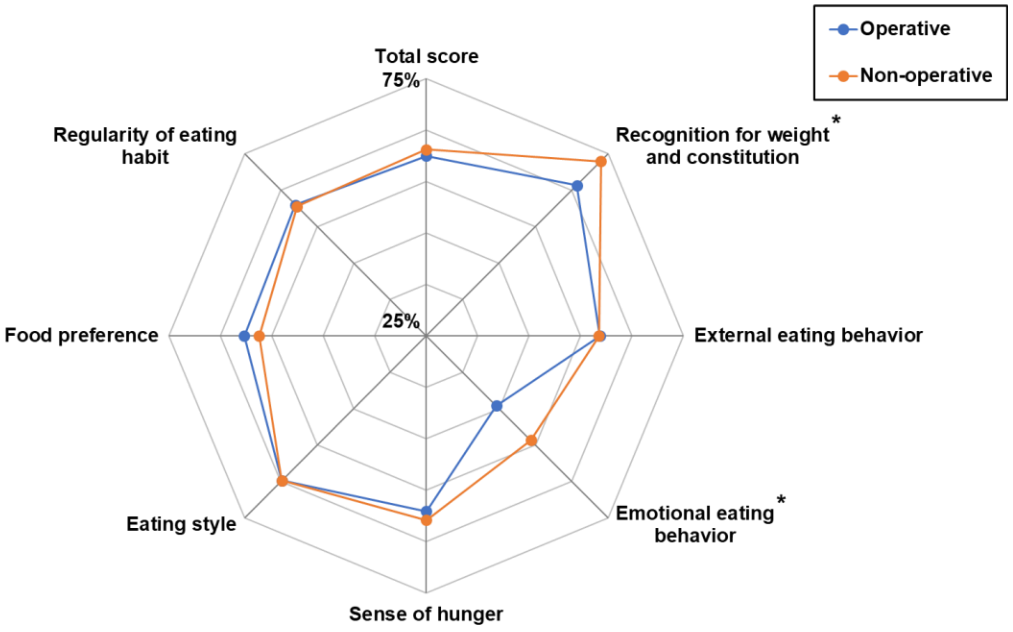

Figure S1

Figure S1. Differences in eating behaviors at the first visit between patients who underwent metabolic and bariatric surgery and those who did not. The scores for eating behaviors in each group were expressed in a radar chart. Significant differences between the groups were found in the "recognition for weight and constitution" and "emotional eating behavior". *$p < 0.05$ (unpaired t-tests).
